# The role of neopterin in patients with primary and metastatic breast cancer: correlation with clinicopathological data

**DOI:** 10.1186/2051-1426-2-S3-P147

**Published:** 2014-11-06

**Authors:** Soheir Rizk Demian, Mona Hamdy El-Sayed, Amal Sobhy El-Sedfy, Ahmed Saad Ahmed, Fadwa Mohammed Ahmedai

**Affiliations:** 1 Medical Research Institute - Alexandria University, Alexandria, Egypt

## 

Breast cancer is the commonest malignancy of women in many parts of the world and comprises 18% of all female cancers. There is one million new cases in the world each year. Neopterin which is a pteridine produced by activation of monocytes by IFN-γ may be used as a marker of immune system activation. The biological function of neopterin is still largely unknown and certain patho-physiologic roles have only recently been elucidated. Therefore, the aim of this work was to investigate the role of neopterin in females with primary and metastatic breast cancer. The obtained results were correlated with different clinicopathological data of those patients. The study population included females with primary and metastatic breast cancer as well as healthy age matched females were taken as a control group. Peripheral blood mononuclear cells were separated from each subject under study by Ficoll-Hypaque density gradient centrifugation technique. The isolated cells were cultured with and without PHA and neopterin levels were measured in serum and culture supernatants by ELISA technique. The results showed that neopterin levels were lowest in serum, followed by culture supernatants without PHA and highest in culture supernatants with PHA in each studied group. The mean neopterin values in serum and culture supernatants with PHA of metastatic breast cancer patients were significantly higher than in the primary breast cancer patients and the control group. Our results also indicated that neopterin levels positively correlated with the tumor grade, tumor stage and the presence of metastasis. From all mentioned data it is clear that, measurement of neopterin either in serum or in culture supernatants with or without PHA can give an indication about immunological status of breast cancer patients' response to therapy and early detection of metastasis. It gives an important and valuable prognostic information before surgical intervention is performed.

